# High Temperature Induced Anthocyanin Inhibition and Active Degradation in *Malus profusion*

**DOI:** 10.3389/fpls.2017.01401

**Published:** 2017-08-09

**Authors:** Rana Naveed Ur Rehman, Yaohua You, Lei Zhang, Bachir Daoura Goudia, Abdul Rehman Khan, Pengmin Li, Fangwang Ma

**Affiliations:** ^1^State Key Laboratory of Crop Stress Biology for Arid Areas, College of Horticulture, Northwest A&F University Yangling, China; ^2^State Key Laboratory of Crop Stress Biology for Arid Areas, College of Agronomy, Northwest A&F University Yangling, China; ^3^Nuclear Institute for Agriculture and Biology Faisalabad, Pakistan

**Keywords:** anthocyanin inhibition, cyanidin 3-galactoside degradation, *Malus* crabapple, peroxidase genes, thermal stress

## Abstract

The red fleshed fruits of *Malus profusion* represent gradual color loss during high temperature in summer, potentially due to active degradation of anthocyanin. The objective of this study was to examine both physiological and molecular evidence of anthocyanin degradation. *Malus* crabapple fruits were exposed to either room temperature (RT = 18 ± 2°C: 25 ± 2°C) or high temperature (HT = 33 ± 2°C: 25 ± 2°C) regimens (12 h: 12 h) under hypoxic (2%) or normoxic (21%) oxygen levels. The results showed that the concentration of cyanidin 3-galactoside (cy-3-gal) was dramatically reduced following HT treatments due to a significant down-regulation of anthocyanin biosynthetic genes (*MpCHS*, *MpDFR*, *MpLDOX*, *MpUFGT*, and *MpMYB10*). Among other repressor MYBs, *MpMYB15* expression was high following HT treatment of the fruit. HT led to the generation of a substantial concentration of H_2_O_2_ due to enhanced activities of superoxide dismutase (SOD), methane dicarboxylic aldehyde (MDA) content and cell sap pH value. Similarly, transcript levels of *MpVHA-B1* and *MpVHA-B2* were reduced which are involved in the vacuolar transportation of anthocyanin. The enzymatic degradation of anthocyanin was eventually enhanced coupled with the oxidative activities of peroxidase (POD) and H_2_O_2_. Conversely, the RT treatments potentially enhanced anthocyanin content by stabilizing physiological attributes (such as MDA, H_2_O_2_, and pH, among others) and sustaining sufficient biosynthetic gene expression levels. Quantitative real-time PCR analysis indicated that the transcription of *MpPOD1*, *MpPOD8* and *MpPOD9* genes in fruit tissues was up-regulated due to HT treatment and that hypoxic conditions seems more compatible with the responsible POD isoenzymes involved in active anthocyanin degradation. The results of the current study could be useful for understanding as well as elucidating the physiological phenomenon and molecular signaling cascade underlying active anthocyanin degradation in *Malus* crops.

## Introduction

Anthocyanins are colored pigments that are ubiquitous in plants and often transitorily accumulated in the peripheral layer of mesophyll tissues (Steyn et al., 2002). Their presence is mainly pertinent to a wide range of functions at the cellular level, like the beautiful coloration of the flower/fruit parts for pollinator recruitment or seed dispersal, photo-protection, thermo-tolerance, and aposematic defense warnings against herbivores (Hughes et al., 2013). Moreover, such pigments are considered potential sources of nutraceuticals due to their pronounced scavenging capacity of reactive oxygen species (ROS) (Agati et al., 2012). Recently, they also have been applied extensively for anticancer, anti-inflammatory and cardiovascular protection.

Generally, fruit color reddening is considered a quality signature of enhanced antioxidant capacity and demonstrates good marketing potential; hence, fruit bagging is accredited among commercial apple cultivars to induce sensitivity to anthocyanin pigment biosynthesis (Zhang et al., 2014). The biosynthesis of anthocyanin occurs via an intricate phenylpropanoid pathway which is predominantly controlled by a plethora of structural genes [such as chalcone synthase (*CHS*), dihydroflavonol 4-reductase (*DFR*), leucoanthocyanidin oxygenase (*LDOX*), and UDP-glucose: flavonoid 3-O-glucosyltransferase (*UFGT*), among others] and a ternary complex of transcription factors (TFs) known as MYB-bHLH-WD40 (MBW) (Feng et al., 2013; Albert et al., 2014). Among the MBW complex, MYB (specifically R2R3 MYBs like *MdMYB1/10*) is the most abundant class and is known to play a paramount role in anthocyanin pigment biosynthesis (Jiang et al., 2014). MYB TFs are induced by light and cold temperature, and their constitutive expression results in the persistent ability to bind to their own promoter in an auto-regulatory loop due to the presence of an extra five tandem repeats, resulting in increased red coloration (Espley et al., 2009; Albert et al., 2014).

Recently, R2R3 repressor MYB TFs have also been reported that inhibit anthocyanin biosynthesis, such as *FaMYB1* (*Fragaria* sp.) (Aharoni et al., 2001), *MdMYB15*, *MdMYB16* (*Malus domestica*) (Lin-Wang et al., 2011) and *MtMYB2* (*Medicago truncatula*) (Jun et al., 2015).

Following by the biosynthesis of anthocyanin pigments, these are transported into the vacuole by the H^+^ pumping activities of H^+^-ATPase (V-ATPase or VHA) and H^+^-pyrophosphatase (V-PPase or VHP). VHA is a multi-subunit and highly conserved proton pump that uses the energy of ATP and shows widespread expression among mature fruits (Hu et al., 2016). However, the VHP pump constitutes a single polypeptide chain, uses the phosphoanhydride bond of pyrophosphate (PPi) as its energy source, and has been extensively reported among young fruits (pear and grape) (Etienne et al., 2002, 2013; Venter et al., 2006). As fruit matures, the activities of VHP protein are greatly reduced due to the plentiful availability of ATP molecules (Etienne et al., 2013). Recently, Hu et al. (2016) demonstrated that *MdVHA-B1* and *MdVHA-B2* (subunits of VHA) play dominent roles in the transportation of anthocyanins and malates into vacuoles.

Previously, many reports have shown that high temperature hampers anthocyanin biosynthesis by suppressing the molecular signaling cascade and leads to a loss of biosynthetic capacity (Ubi et al., 2006; Movahed et al., 2016). However, the loss of biosynthetic capacity (only) does not confer active degradation of anthocyanin pigments. Instead, it is distinguished enzymatic and systematic process (Vaknin et al., 2005) that might differ among various crop species. Similarly, POD enzyme has been reported to be involved in the active degradation of anthocyanin in *Brunfelsia calycina* flowers (Zipor et al., 2015). However, comprehensive details regarding the active degradation of anthocyanin among deciduous fruits (like *Malus* sp.) remain largely unknown.

*Malus profusion* is a medium-sized tree that is grown extensively in semi-arid temperate zones of China and produces small red-fleshed fruits (<5 cm) that are sometimes used for traditional medicinal purposes. During summer, due to the high temperature (≥30°C), the fruit exhibits a gradual loss of color due to concurrent turnover of anthocyanin pigments. However, molecular and physiological evidence explaining this phenomenon remain obscure. Abiotic stress like high temperature triggers the production of ROS, and hence the enzymatic antioxidant system (SOD, POD, among others) is used to protect other cellular organelles against its deleterious effects (Mittler, 2002). However, consistently elevated ROS (like H_2_O_2_ or $O_{2}^{•-}$) can damage membrane integrity and disturb many other biological processes (Ali et al., 2016).

Taken together, these findings indicate that active degradation of anthocyanin is initiated due to loss of biosynthetic capacity and the beginning of specific enzymatic degradation reactions. In the former case, loss of activity of biosynthetic enzymes/gene expression or activation of the repressor MYBs predominate, while enzymatic degradation is induced due to specific enzymes (like POD, or polyphenol oxidase). Rationally, class III POD seems to be an active candidate due to its co-localization with anthocyanin in the vacuole (Zipor et al., 2015). However, excluding a few reports such as (Movahed et al., 2016), molecular evidence of POD is still lacking due to post-translational modifications and the formation of divergent isoenzymes. In addition, Awad and de Jager (2000) found that low oxygen levels have no effect on anthocyanin biosynthesis; however, the effect of hypoxic conditions on the rate of active degradation has not been assessed before.

Fruit color is a primary determinant of the consumer’s choice, but very few studies have examined gradual color loss. In the present study, we assessed both physiological and molecular evidence of the active degradation of anthocyanin among fruits of *Malus profusion* under hypoxic and normoxic oxygen levels. This information will be useful for understanding the mechanism of pigment degradation in *Malus* fruits during high temperature in summer.

## Materials and Methods

### Plant Materials and Treatments

In this study, fruits were harvested from mature trees (8- to 10-years-old) of *Malus profusion* having a central leader growth habit with a planting distance of 3 × 4 m located at Northwest A&F University, Pomology experimental area (34°N, 108°E) Yangling, Shaanxi, China. The trees were receiving standard management practices (such as irrigation, fertilization, and pruning) during 2016. Red-fleshed fruits of *Malus profusion* were collected at physiological maturity 110–115 days after blooming (DAB). After washing and drying at ambient temperature (25 ± 2°C), the fruits were thoroughly randomized and divided into five homogenous groups (treatments). Peel and flesh (together) of one group of fruits (0 day) was stored immediately at -80°C. While rest of treatments were exposed to two temperatures [high temperature (HT) and room temperature (RT)] with an alternative temperature regime of (12: 12 h) under two oxygen levels viz; HT-2% = HT 33 ± 2°C: 25 ± 2°C 2% O_2_; HT-21% = HT 33 ± 2°C: 25 ± 2°C + 21% O_2_; RT-2% = RT 18 ± 2°C: 25 ± 2°C + 2% O_2_; RT-21% = RT 18 ± 2°C: 25 ± 2°C + 21% O_2_ at 85 ± 5% RH; for 1 week in the dark. The 2 and 21% O_2_ was provided by modified packaging in plastic bags and a continuous flow-through system, respectively, in temperature controlled chamber. After 1 week, the peel and flesh (together) were ground into a powder in liquid nitrogen using the A11 blender (IKA^®^ Works, Pittsburgh, PA, United States) and immediately stored at -80°C.

### The pH, H_2_O_2_ and MDA Contents

The pH values of fresh fruit were measured as reported previously (Vaknin et al., 2005). The fruit (1 g) was ground in 10 ml deionized water using a hi-speed dispersant (XHF-D, Shanghai, China), and the pH was determined immediately using a Mettler Toledo pH meter (FE20, Shanghai, China).

The H_2_O_2_ concentration was assayed according to the method of Zhang et al. (2014) and expressed as μmol kg^-1^ FW (fresh weight). Briefly, frozen samples were ground in 5% (w/v) trichloro-acetic acid (TCA) cold extraction buffer followed by centrifugation at 13,000 × *g* for 20 min at 4°C. The supernatant was neutralized to pH 7.8 with NH_4_OH, and the sample was divided into two 500 μl aliquots (A and B). Twenty units of catalase (CAT, EC 1.11.1.6) were added to aliquot ‘A.’ Both A and B were kept at 20°C for 10 min, followed by the addition of 500 μl colorimetric reagent. The colorimetric reagent was freshly prepared by mixing 0.3 mM potassium titanium oxalate and 0.3 mM 4-(2-pyridylazo) resorcinol monosodium salt and water at a ratio of 1:1:2 (v/v). The assay mixture was incubated at 45°C for 20 min before determining the absorbance at 508 nm using a UV–vis spectrophotometer (2450 Shimadzu, Tokyo, Japan).

The MDA content was determined as previously described (Xue et al., 2015) after minor modifications and expressed as the μmol g^-1^ FW. Frozen samples were ground in buffer containing ethanol: water (80: 20 v/v), and centrifuged at 5,000 × *g* for 10 min at 4°C. The supernatant (1 ml) was mixed vigorously with 0.65% thiobarbituric acid (in 10% TCA), heated at 95°C until boiling and promptly cooled on ice and centrifuged at 3,500 × *g* for 2 min to remove the flocculate. The clear supernatant was used to determine the absorbance at 450, 532, and 600 nm using a UV–vis spectrophotometer (2450 Shimadzu, Tokyo, Japan).

### Anthocyanin Quantification

Anthocyanin was quantified by high-performance liquid chromatograph (HPLC) as described previously (Zhang et al., 2014). Briefly, frozen fruit tissues were ground using phenolic extract solution containing methanol: formic acid (70%: 2%), centrifuged at 12,500 × *g* for 20 min at 4°C and the supernatant was filtered using a syringe filter (0.45 μm) prior to HPLC injection (HP1200 Shimadzu, Tokyo, Japan). The HP1200 was equipped with a diode array detector (Agilent technologies, Palo Alto, CA, United States), an Inertsil ODS-3 Guard Column (4.0 mm × 10 mm; 5.0 μm particle size; GL Sciences, Tokyo, Japan), and an Inertsil ODS-3 column (4.6 mm × 250 mm; 5.0 μm particle size; GL Sciences, Tokyo, Japan). Solvent A and B contained 10% formic acid in water and 10% formic acid in HPLC-grade acetonitrile, respectively. The order of the gradient used was as follows: 95% A (0 min), 85% A (25 min), 78% A (42 min), 64% A (60 min), and 95% A (65 min). The flow rate was 1.0 ml min^-1^ at 30°C, the post-run time was 10 min, and monitoring was performed at 520 nm for cy-3-gal. The cy-3-gal peak was identified by comparing retention times and UV spectra with reliable standards. The concentration was determined based on the peak area and calibration curves derived from standards of the corresponding compound. All phenolic standards were obtained from Sigma–Aldrich (St. Louis, MO, United States), ExtraSynthese (Genay, France), and AApin Chemicals (Abingdon, Oxon, United Kingdom).

### Enzyme Assays

Frozen samples of *Malus profusion* were ground in extraction buffer containing 0.014 M β-mercaptoethanol, 0.002 M EDTA-Na_2_, 0.5% Triton X-100, 1% BSA, 0.005 M DDT, 10% glycerol and 0.1 M Tris–HCl (pH 7.5). After thorough homogenization, the samples were centrifuged at 12,000 × *g* for 15 min at 4°C. The supernatant was quickly used for further analysis.

The activity of peroxidase (POD; EC 1.11.1.7) was determined by guaiacol oxidation according to Ali et al. (2016) with slight modifications and expressed as μmol m^-2^ s^-1^. The 1 ml reaction mixture contained 0.05 M sodium phosphate buffer (pH 7.0), 0.01 M H_2_O_2_, 0.01 M guaiacol and 10 μl of the enzyme extract. The reaction was initiated by adding H_2_O_2_, and the activity was determined by monitoring the change in absorbance at 470 nm using a UV-Vis spectrophotometer (2450 Shimadzu, Tokyo, Japan).

The activity of superoxide dismutase (SOD; EC 1.15.1.1) was determined by monitoring the inhibition of the photochemical reduction of nitroblue tetrazolium (NBT) according to previously described methods (Štajner and Popović, 2009) and expressed as 10^5^ U kg^-1^ FW. The 1 ml reaction mixture contained 580 μl of Tris–HCl pH 7.5 (0.1 M), 100 μl of methionine (0.065 M), 100 μl of NBT (50 μM), 200 μl of riboflavin (20 μM), and 20 μl of the enzyme extract. The test tubes wrapped in aluminum foil were vigorously mixed and irradiated for 30 min under fluorescent light before determining the absorbance at 560 nm using a UV-Vis spectrophotometer (2450 Shimadzu, Tokyo, Japan).

### Quantitative Real-time PCR Analyses

Fruit RNA was extracted using the SDS-phenol method according to Zhang et al. (2014) and quantitated using a NanoDrop 2000 micro-volume spectrophotometer (Thermo Fisher Scientific, Waltham, MA, United States) at 260 and 280 nm. RNA quality and integrity were assessed by 1.2% agarose gel electrophoresis, and the gel was stained with ethidium bromide. First-strand cDNA was synthesized from 1 μg of total RNA using the reverse PrimeScript RT reagent Kit^TM^ (Takara, Dalian, China) according to the manufacturer’s instructions. cDNA amplifications were carried out with primers for *MpCHS*, *MpDFR*, *MpLDOX*, *MpUFGT*, *MpMYB10*, *MpMYB15*, *MpMYB16 MpMYB111*, *MpPOD1*, *MpPOD21*, *MpPOD40*, *MpPOD01-1*, *MpPOD8*, *MpPOD9*, *MpVHA-B1*, and *MpVHA-B2* (Supplementary Table S1). All reactions were performed with three biological replicates containing 10 μl of SYBR Green MasterMix (SYBR Premix EX Taq^TM^, Dalian, China), 2 μl of cDNA, 6.4 μl of nuclease-free water, and 0.8 μl of each primer to a final volume of 20 μl. The Q5 Multicolor Real-Time PCR Detection System (Bio-Rad Laboratories, Hercules, CA, United States) was used for gene quantification. *MpActin* was used to standardize the cDNA samples for different genes (Kumar and Singh, 2015), and hence the results are presented as the normalized relative expression. The relative expression levels of target genes were calculated according to the 2^-ΔΔ*C*_T_^ method (Zhang et al., 2014).

### Gene Selection and Phylogenetic Analyses

**FIGURE 1:**
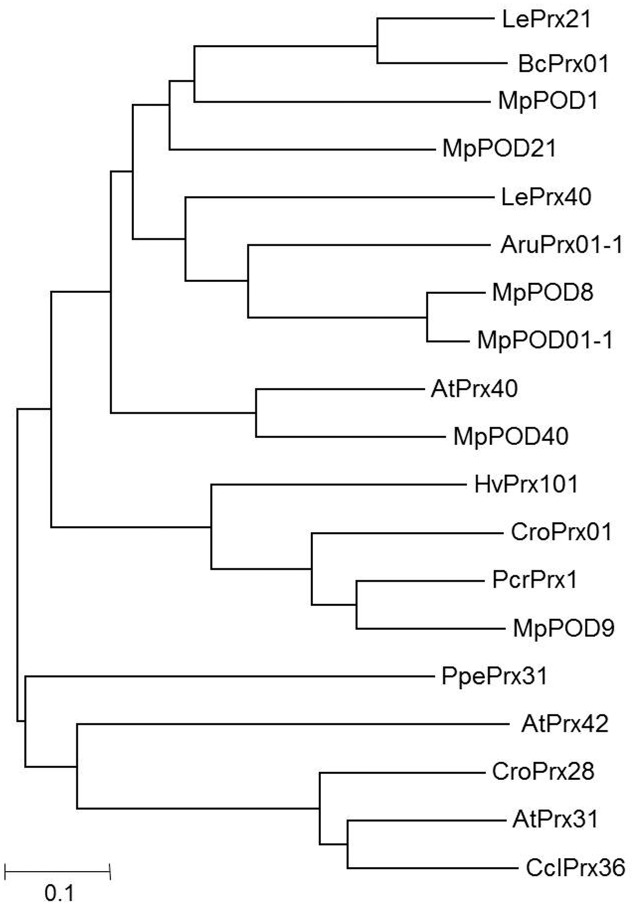
Neighbor-joining phylogenetic tree. The following protein sequences retrieved from class III PeroxiBase (http://peroxibase.toulouse.inra.fr/index.php) were used: *LePrx21* (*Lycopersicon esculentum*), *BcPrx01* (*Brunfelsia calycina*), *LePrx40* (*Lycopersicon esculentum*), *AruPrx01-1* (*Armoracia rusticana*), *AtPrx31*, *AtPrx40*, *AtPrx42* (*Arabidopsis thaliana*), *HvPrx101* (*Hordeum vulgare*), *CroPrx01* (*Catharanthus roseus*), *PcrPrx1* (*Petroselinum crispum*), *PpePrx31* (*Prunus persica*), *CroPrx28* (*Catharanthus roseus*), *CclPrx36* (*Citrus clementina*), *MpPOD1* (MDP0000629636), *MpPOD21* (MDP0000209189), *MpPOD8* (MDP0000208152), *MpPOD01-1* (MDP0000706473), *MpPOD40* (MDP0000211003), *MpPOD9* (MDP0000176436).

The nucleotide sequences of *MpCHS*, *MpDFR*, *MpLDOX*, *MpUFGT*, *MpMYB10*, *MpMYB15*, *MpMYB16 MpMYB111*, *MpVHA-B1*, and *MpVHA-B2* were obtained from the *Malus* genome database^1^. Similarly, the protein sequences of class III POD among *Malus* sp. (*MpPOD21*, *MpPOD40*, *MpPOD01-1*, *MpPOD8*, and *MpPOD9*) were retrieved from PeroxiBase^2^. A BLAST analysis of the protein sequences was performed using the *Malus* genome databases^3^, and nucleotide sequences were obtained. For the nucleotide sequence of *MpPOD1*, the closest homologs of the protein sequences of *BcPrx01* were selected from the PeroxiBase database and applied for a BLAST search against the *Malus* genome database. For phylogenetic tree construction of POD genes, the MUSCLE program was used with default parameters (**Figure 1**).

### Statistical Analysis

All data were subjected to analysis of variance with SPSS 16.0 for Windows (SPSS Inc., Chicago, IL, United States). Data were analyzed using one-way analysis of variance (ANOVA) followed by the least significant difference (LSD) computed at *P* < 0.05. Five biological replicates for all physiological parameters (*n* = 5) and three for qPCR (*n* = 3) were used for all statistical analysis.

## Results

### High Temperature Reduced Anthocyanin Content

**FIGURE 2:**
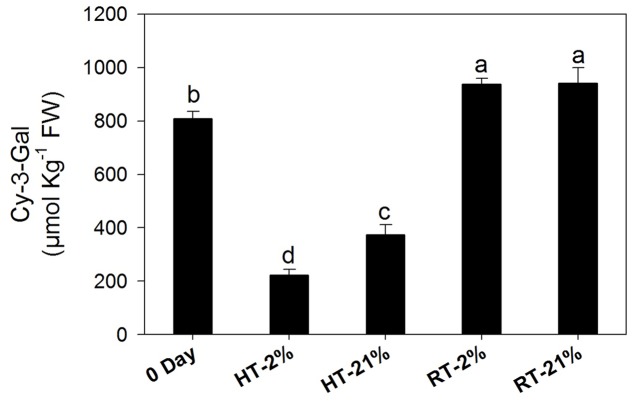
Amount of cy-3-gal among different temperature-treated fruits. Each bar shows the mean ± SE (*n* = 5). Lowercase letters above the bars indicate significantly different values among different temperature-treated fruits (*P* < 0.05). HT or RT represents high or room temperature under two oxygen levels (2 or 21%).

Cy-3-gal was identified as main anthocyanin pigment by HPLC analysis. The concentration of cy-3-gal was markedly (*P* < 0.05) diminished under hypoxic (67%) and normoxic (54%) high-temperature treatments, while RT treatments showed significant biosynthesis of cy-3-gal regardless of the oxygen level (**Figure 2**).

### High Temperature Induced Down-regulation of Biosynthetic Genes and Epistatic Gene Interactions with Repressor MYBs

**FIGURE 3:**
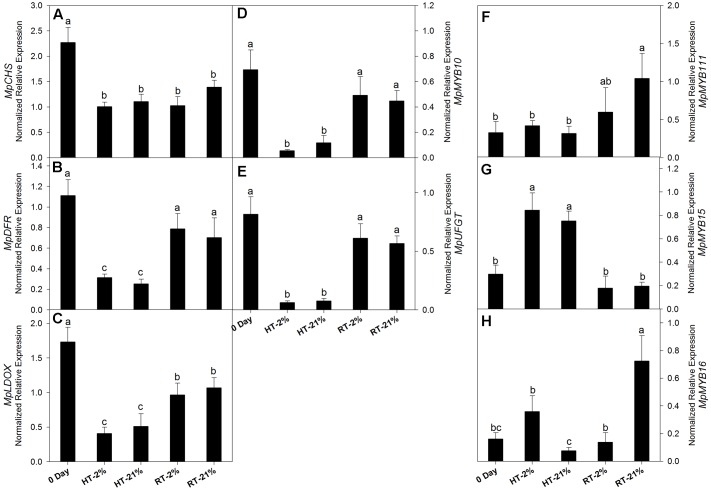
Relative transcription levels of biosynthetic genes and repressor MYBs. *MpCHS* **(A)**, *MpDFR* **(B)**, *MpLDOX* **(C)**, *MpMYB10* **(D)**, *MpUFGT* **(E)** and repressor MYBs *MpMYB111* **(F)**, *MpMYB15* **(G)** and *MpMYB16* **(H)** among different temperature-treated fruits. Each bar is the mean ± SE (*n* = 3). Lowercase letters above the bars indicate significantly different values among different temperature treated fruits (*P* < 0.05). HT or RT represents high or room temperature under two oxygen levels (2 or 21%).

Cy-3-gal is a product of the phenylpropanoid pathway. To evaluate the biosynthetic potential among different treatments, the transcriptional level of key genes and repressor *MYBs* were assessed (**Figures 3A–H**). In general, relative gene expressions for all the putative biosynthetic genes were tremendously high on day 0. After fruit detachment from the tree, HT (2/21% O_2_ on average) treatments exhibited a significant (*P* < 0.05) down-regulation of *MpCHS* (twofold), *MpDFR* (fourfold), *MpLDOX* (3.8-fold), *MpUFGT* (12-fold) and *MpMYB10* (ninefold) (**Figures 3A–E**). However, the expression levels were maintained by 50–70% (on average) for the RT treatments.

In the case of repressor MYBs like *MpMYB111*, *MpMYB15* and *MpMYB16* (**Figures 3F–H**), not all of them correlated with the loss of biosynthetic capacity. However, *MpMYB15* showed an integrative higher expression level (*P* < 0.05) at HT under hypoxic (2.8-fold) and normoxic (2.5-fold) conditions (**Figure 3G**).

### Role of Other Physiological and Molecular Attributes at High Temperature

**FIGURE 4:**
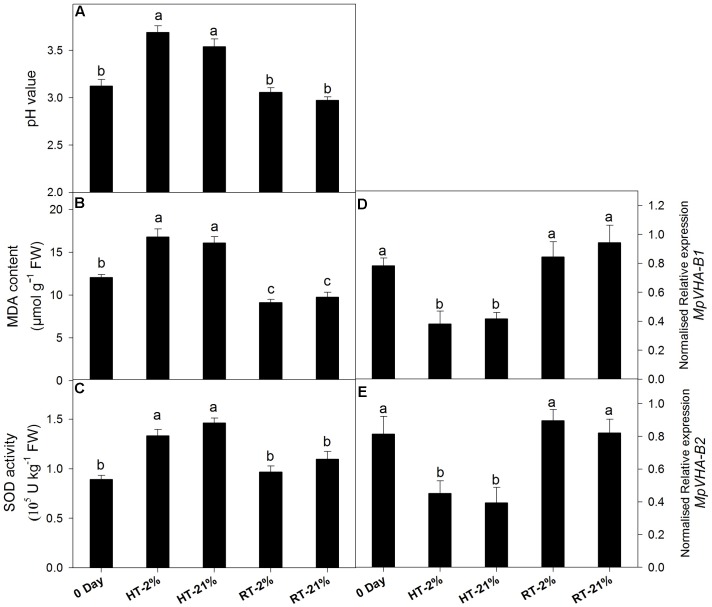
Effect of hypoxic thermal stress on other physiological events. The pH value **(A)**, MDA content **(B)**, SOD activity **(C)** and relative transcript levels of *MpVHA-B1* **(D)** and *MpVHA-B2* **(E)** among different temperature-treated fruits. For **(A–C)** and **(D,E)** each bar is the mean ± SE (*n* = 5) and (*n* = 3), respectively. Lowercase letters above the bars indicate significantly different values among different temperature-treated fruits (*P* < 0.05). HT or RT represents high or room temperature under two oxygen levels (2 or 21%).

A loss of anthocyanin biosynthetic potential and inhibition by repressor MYBs was observed for the HT treatments. To assess the physiological phenomenon regarding remaining the cy-3-gal concentration in the vacuole, we quantified the MDA content, SOD activity, pH value, and transcription levels of the sub units of vacuolar pump (*MpVHA-B1* and *MpVHA-B2*) (**Figures 4A–E**).

High temperature treatment showed an augmented MDA content (1.5-fold) and pH values (1.15-fold) (**Figures 4A,B**). Transcript levels of *MpVHA-B1* and *MpVHA-B2* were also significantly (*P* < 0.05) lower for HT treatments compared with 0 day or RT treatments (**Figures 4D,E**). However, the RT treatments showed significantly (*P* < 0.05) reduced MDA contents and inconsistent variations in pH values. The activity of SOD was significantly (*P* < 0.05) increased for HT treatment (**Figure 4C**).

### Active Enzymatic Degradation of Anthocyanin

**FIGURE 5:**
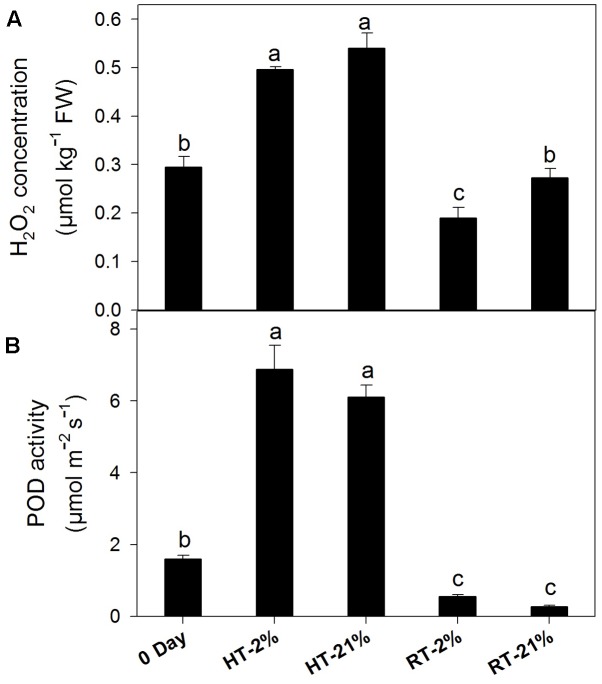
Active degradation of anthocyanin among different temperature-treated fruits. H_2_O_2_ content **(A)** and POD activity **(B)** lead to coupled oxidation reactions with anthocyanin. Each bar is the mean ± SE (*n* = 5). Lowercase letters above the bars indicate significantly different values among different temperature-treated fruits (*P* < 0.05). HT or RT represents high or room temperature under two oxygen levels (2 or 21%).

Abiotic stress triggers H_2_O_2_ production, which together with POD can undergo coupled oxidation with anthocyanin. H_2_O_2_ was higher for heated fruits and significantly reduced (*P* < 0.05) for hypoxic RT (**Figure 5A**). Similarly, HT treatments showed elevated (*P* < 0.05) enzymatic activity regardless of the oxygen level (average of fourfold), while it remained at less than half of the original level for RT treatments (**Figure 5B**).

**FIGURE 6:**
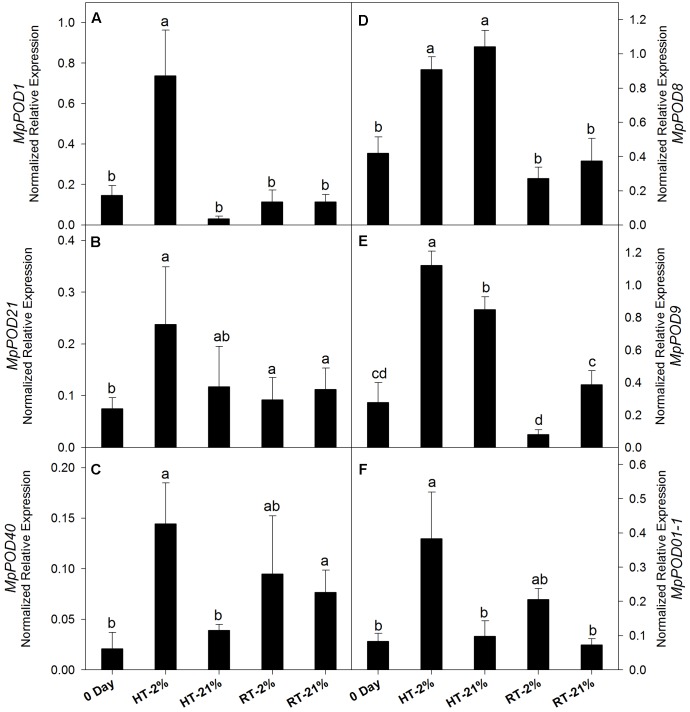
Relative expression levels of POD genes among different temperature-treated fruits. Transcription level of *MpPOD1* **(A)**, *MpPOD21* **(B)**, *MpPOD40* **(C)**, *MpPOD8* **(D)**, *MpPOD9* **(E)** and *MpPOD01-1* **(F)** genes. Each bar is the mean ± SE (*n* = 3). Lowercase letters above the bars indicate significantly different values among different temperature-treated fruits (*P* < 0.05) calculated using one-way analysis of variance (ANOVA) followed by the least significant difference (LSD). HT or RT represents high or room temperature under two oxygen levels (2 or 21%).

The expression levels of different POD genes are presented in (**Figures 6A–F**). *MpPOD8* and *MpPOD9* showed higher (*P* < 0.05) expression in response to HT treatment (**Figures 6D,E**). In contrast, *MpPOD1*, *MpPOD21*, *MpPOD40*, and *MpPOD01-1* exhibited relatively high transcript levels for only hypoxic HT (**Figures 6A–C,F**). However, transcription levels remained non-consistent for RT treatments except *MpPOD9* which shows significant (*P* < 0.05) variation at different oxygen levels (**Figure 6E**).

## Discussion

Immense literature is available for anthocyanin biosynthesis, but very few studies have assessed active degradation among *Malus* species. In the current study, we have provided physiological and genetic evidences of color loss in *Malus profusion* fruit.

Concentration of anthocyanin was highly decreased following HT treatment due to the thermal sensitivity of *MpMYB10* and late biosynthetic genes (*MpDFR*, *MpANS*, and *MpUFGT*), as previously reported (Steyn et al., 2004; Ubi et al., 2006). Regarding the RT treatments, anthocyanin accumulation was significantly enhanced, while the transcript levels of biosynthetic genes were not significant at 0 day (**Figures 3B,D,E**). This phenomenon might be due to improved pigment stability at room temperature, since in orchards, fruits are exposed to abbreviated heat stress (nearly 4 h) at noon and a net anthocyanin at day 0 counterbalance by simultaneous biosynthesis and active degradation. However, after RT exposure, the rate of anthocyanin accumulation was significantly enhanced, indicating that the stability of anthocyanin plays an inevitable role in the overall pigment content (Shaked-Sachray et al., 2002; Sinilal et al., 2011).

Our results corroborated the findings of Lin-Wang et al. (2011) showing that *MpMYB15* functioned well in fruit at a high temperature (**Figure 3G**). We also speculate that *MpMYB111*/*MpMYB16* (**Figures 3F,H**) have higher expression levels for RT treatments (unheated), while the concentrations of anthocyanin are not as markedly decreased. Although the interplay between biosynthetic or repressor MYBs has not been completely understood, these results suggest that biosynthetic MYB play a dominant role over repressor MYB under favorable conditions, which might be due to epistatic interactions between the MBW complex and repressor MYB, relating to functional congruency or redundancy and resulting in phenotypic plasticity (Lightbourn et al., 2007; Nakatsuka et al., 2013).

**FIGURE 7:**
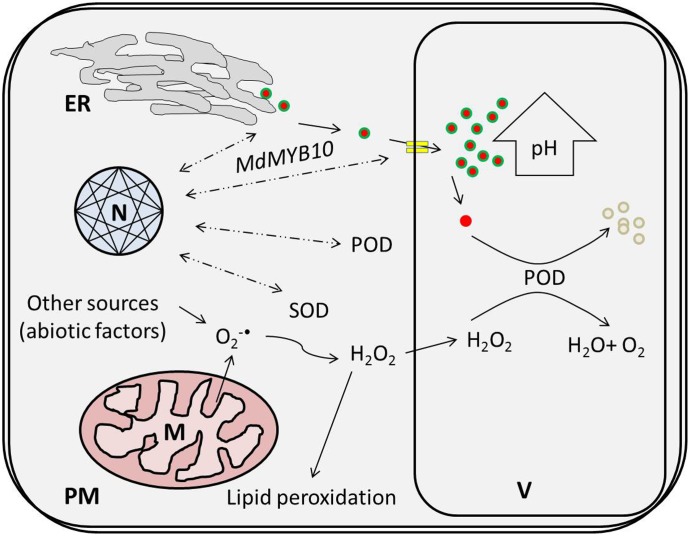
High temperature-induced physiological changes at the cellular level lead to the active degradation of cy-3-gal. ROS produced via thermal induction leads to a coupled oxidative reaction with POD and phenolic in the vacuole. ER, N, V, M, PM, POD, and SOD represent the endoplasmic reticulum, nucleus, vacuole, plasma membrane, peroxidase, and superoxide dismutase, respectively.

Regarding physiological features, the concentration of H_2_O_2_ increased in response to the HT treatments, which might have been due to mitochondria, which represent a predominant site of ROS production among non-photosynthetic tissues in the dark (Navrot et al., 2007). In mitochondria, dysfunctional cytochrome c oxidase, as an essential component of the electron transfer chain (ETC), has been associated with superoxide ($O_{2}^{•-}$) free radical generation (due to attack of excessive ROS) and is considered to confer extreme deleterious effects on other biological activities (Sedlák et al., 2010; Shafi et al., 2015). Enhanced SOD activity in response to HT treatments supports the hypothesis that $O_{2}^{•-}$ are promptly scavenged to H_2_O_2_ (being less deleterious) (Navrot et al., 2007) and that this consistent increment in H_2_O_2_ concentration leads to membrane oxidation like lipid peroxidation, disruption of enzymatic inhibition and DNA/RNA damage (**Figure 7**) (Mittler, 2002). Hence, the MDA content was improved nearly 1.5-fold for the HT treatments in comparison to 0 day.

Moreover, the expression levels of *MpVHA-B1* and *MpVHA-B2* decreased in response to HT treatment, suggesting that vacuolar transportation of anthocyanin is dramatically reduced. Interestingly, three isoforms of VHA protein are present in apple, which are encoded by three genes (*MpVHA-B1*, *MpVHA-B2*, and *MpVHA-B3*) containing MYB recognition sites in their promoter regions. By using EMSA, ChIP-PCR and GUS assays, Hu et al. (2016) demonstrated that *MdMYB1/10* only binds to *MdVHA-B1* and *MdVHA-B2* (not *MdVHA-B3*) to facilitate the coupled transportation of malate and anthocyanin into the vacuole. However, reduced expression levels of *MpVHA-B1* and *MpVHA-B2* are a hallmark of perturbed transportation of anthocyanin into the vacuole.

In addition, PODs are multifunctional enzymes, and their thermal induction has been well proposed to provide protection against H_2_O_2_ during stress conditions in apple (Requesens et al., 2014). Similarly in strawberry fruits, enhanced POD activity is supposed to be associated with membrane damage recovery against gradual heat stress (Gulen and Eris, 2004). Higher plants typically contain 8–15 kinds of POD families and are generally classified based on their isoelectric points (acidic or basic) isoenzymes (Pearse et al., 2005). Among those families, the role of class III POD (basic) has been well demonstrated and characterized during the active degradation of anthocyanin (Vaknin et al., 2005; Zhang et al., 2005; Zipor et al., 2015). However, it is difficult to obtain precise molecular evidence about class III POD genes involved in the active degradation of anthocyanin due to multigene family control and the formation of divergent isoenzymes during post-transcriptional modifications (Movahed et al., 2016). In the present study, hypoxic high temperature induced PODs (*MpPOD01-1*, *MpPOD9*, and *MpPOD1*) have been elucidated. Their induction might be regulated by promoter sensitivity of several abiotic stresses. The promoters of different abiotic stress-responsive genes have varying number of regulatory elements that enhance the expression profile of certain genes, such as LTRE (low temperature-responsive element) or ABRE (abscisic acid-responsive element) are the key stimulant for regulating stress inducible gene expressions (Bhuria et al., 2016). These abiotic stresses (thermal, dehydration or others) independently or together co-regulate the promoters of stress responsive genes. Induction of specific POD genes might be due to the coupled effect of hypoxic conditions and thermal stress since ultra-low oxygen storage at cold temperature have negligible effects on the biosynthesis of anthocyanin and other phenolic compounds (Awad and de Jager, 2000). Another possible reason for the low anthocyanin content in hypoxic thermal conditions may be attributed to the disturbed respiratory quotient, resulting in the production of excessive acetaldehyde (or other similar compounds) during anaerobic respiration, which sequentially affects other metabolic reactions (Thewes et al., 2017).

Finally, thermal stress reduces the biosynthetic potential of anthocyanin and triggers ROS generation. It also induces specific POD genes due to the coupled effect of HT and low oxygen. Enhanced POD and H_2_O_2_ activities induce sequential coupled oxidation of anthocyanin pigment and cause color loss during high temperatures in *Malus* crabapple fruits, as illustrated in **Figure 7**.

## Conclusion

Anthocyanin biosynthesis is inhibited and degraded at high temperatures due to up-regulation of the repressor *MpMYB15* and coupled oxidation reactions of POD and H_2_O_2_, respectively. Vacuolar transportation of anthocyanin pigments was also reduced during thermal stress due to down-regulation of *MpVHA-B1* and *MpVHA-B2*. A lack of information about the MBW complex and repressor *MYB* interplay is a major handicap in distinguishing the loss of anthocyanin biosynthetic capacity and its suppression. However, RT treatments with modified packaging (2 and 21% O_2_) sustained better physiological attributes over the same duration. Further studies should assess the functional characterization of POD genes and their divergent isoenzymes. Such information will be useful for understanding physiological and molecular signaling cascades in active anthocyanin degradation, thus improving the fruit quality and economic traits.

## Author Contributions

All authors made contribution to the experiment work and manuscript write up. RNUR made substantial contribution to the design of the work, acquisition, analysis, and interpretation of data and drafting the manuscript. YY and LZ analyzed data. BDG and ARK revised manuscript. PL and FM made contribution to the design of the work, interpretation of data and critically revision of the manuscript. All authors made contribution to the approval of the final version of the manuscript to be published and agreed to be accountable for all aspects of the work in ensuring that questions related to the accuracy or integrity of any part of the work are appropriately investigated and resolved.

## Conflict of Interest Statement

The authors declare that the research was conducted in the absence of any commercial or financial relationships that could be construed as a potential conflict of interest.

## Footnotes

1 https://www.rosaceae.org/

2 http://peroxibase.toulouse.inra.fr/index.php

3 https://www.rosaceae.org/
