# Supplementary material for: High Temperature Induced Anthocyanin Inhibition and Active Degradation in Malus profusion
Source: Front Plant Sci. 2017 Aug 9;8:1401. doi: 10.3389/fpls.2017.01401 (PMC5552711; doi:10.3389/fpls.2017.01401)
Supplement: Supplementary file 1 [file Data_Sheet_1.docx]

**High temperature induced anthocyanin inhibition and active degradation in *Malus profusion***

Rana Naveed Ur Rehman, Yaohua You, Lei Zhang, Bachir Daoura Goudia, Abdul Rehman Khan, Pengmin Li^*^ and Fangwang Ma

***Correspondence:**

*Corresponding Author*

[lipm@nwsuaf.edu.cn](mailto:lipm@nwsuaf.edu.cn)

**Table S1.** Sequences of the oligonucleotide primers used to study the gene expression.

| **Gene** | **Transcript Name** | **Primer sequence (5′–3′)** |
| --- | --- | --- |
| *MpActin* | MDP0000774288 | F: TGTGCCTGCCATGTATGTT  R: TCACCAGAGTCCAGCACAA |
| *MpCHS* | MDP0000686666 | F: GGAGACAACTGGAGAAGGACTGGAA  R: CGACATTGATACTGGTGTCTTCA |
| *MpDFR* | MDP0000494976 | F: GATAGGGTTTGAGTTCAAGTA  R: TCTCCTCAGCAGCCTCAGTTTTCT |
| *MpLDOX* | MDP0000360447 | F: CCAAGTGAAGCGGGTTGTGCT  R: CAAAGCAGGCGGACAGGAGTAGC |
| *MpMYB10* | MDP0000259614 | F: TGCCTGGACTCGAGAGGAAGACA  R: CCTGTTTCCCAAAAGCCTGTGAA |
| *MpUFGT* | MDP0000478252 | F: CACTTTCTGGATCTCCGGACTCAA  R: CCGAAAATGACTCCTTCCGCTAAG |
| *MpVHA-B1* | MDP0000945182 | F: CTGCAGCACGGTCAAATTA  R: CGCGCACACACACATATACA |
| *MpVHA-B2* | MDP0000631168 | F: TTTGAGGGAAGGAGTGTATGATGG  R: CTAAGAAGCAAGGAGGGAAGAGG |
| *MpPOD1* | MDP0000629636 | F: ACCCAAGAAACAAGAACACCCT  R: AATTCCCATCATATTTTGAAAT |
| *MpPOD01-1* | MDP0000706473 | F: TTAGAAAAATCGATTACCACCT  R: CTCTTGAATGACTCCACGCACA |
| *MpPOD08* | MDP0000208152 | F: AGGCAGTCCTGATCCGACCTTGA  R: CGGGCTTATATTACCCATTTTAA |
| *MpPOD09* | MDP0000176436 | F: AGGGATTCTGTTTTCCTGATGTTTT  R: AAGGTTTGCTAGGGTTGCATTTTGT |
| *MpPOD21* | MDP0000209189 | F: CAATTGTCCTCTACTTTCTATGGAACA  R: GCACCTGCTGTTTTCTCTCCTGT |
| *MpPOD40* | MDP0000211003 | F: CAAAAACAATCGCAACGCCCAG  R: ACTTAAACAGCCAATTACCCCT |
